# Fertility-associated biochemical components in seminal plasma and serum of buffalo (*Bubalus bubalis*) bulls

**DOI:** 10.3389/fvets.2022.1043379

**Published:** 2023-01-17

**Authors:** Essam A. Almadaly, Abdel-Basaer S. Abdel-Salam, Ferial M. Sahwan, Khaled A. Kahilo, Tarek K. Abouzed, Wael B. El-Domany

**Affiliations:** ^1^Department of Theriogenology, Faculty of Veterinary Medicine, Kafrelsheikh University, Kafr El-Sheikh, Egypt; ^2^Laboratory of Medical Complex Hospital, Ministry of Health, Tanta, El-Gharbia, Egypt; ^3^Animal Breeding and Production, Animal Husbandry and Animal Wealth Development Department, Faculty of Veterinary Medicine, Alexandria University, Alexandria, Egypt; ^4^Department of Biochemistry, Faculty of Veterinary Medicine, Kafrelsheikh University, Kafr El-Sheikh, Egypt; ^5^Department of Theriogenology, Faculty of Veterinary Medicine, Mansoura University, Mansoura, Egypt

**Keywords:** antioxidants, buffalo bulls, fertility biomarkers, hormones, proteomic, sperm kinematics

## Abstract

The present study looks for components in seminal plasma (SP) and/or serum that are closely related to *in vivo* fertility of buffalo bulls. Fourteen healthy mature buffalo bulls were classified according to their *in vivo* fertility into fertile (*n* = 10) and subfertile (*n* = 4) groups. Semen and serum samples were collected from all animals for 12 replicates. The collected ejaculates were examined for sperm characteristics before being centrifuged to collect SP for hormonal (FSH, LH, testosterone, and IGF-1), biochemical [total antioxidant capacity (TAC), catalase (CAT), glutathione peroxidase (GPx), nitric oxide (NO), malondialdehyde (MDA), fructose, total protein, albumin, triglycerides, cholesterol, and high-density lipoprotein (HDL)] and proteomic (SDS-PAGE) analyses. Likewise, serum levels of FSH, LH, testosterone, IGF-1, glucose, total protein, albumin, triglycerides, cholesterol, and HDL were determined. All sperm characteristics and the majority of sperm kinematics were (*P* < 0.01) different between fertile and subfertile groups. Seminal and serum levels of FSH, LH, testosterone, and IGF-1 were higher (*P* < 0.01) in the fertile group, but only seminal fructose, total protein, albumin, triglycerides, cholesterol, and HDL were higher (*P* < 0.01) in the fertile group. Moreover, the fertile group had greater TAC, CAT, GPx, and NO, but the subfertile group had greater MDA. Protein bands of 14, 15, 26, 30, and 55 kDa were larger and denser in the SP of the fertile group but were smaller and faint to absent in that of the subfertile group. Also, the protein fractions of detected protein bands demonstrated a substantial influence of fertility on those of 16, 26, 30, and 55 kDa. In conclusion, sperm characteristics and kinematics with serum, and/or seminal hormonal and biochemical components, should be evaluated for reliable prediction of buffalo bull fertility. Furthermore, protein bands of 26, 30, and 55 kDa may represent fertility-associated proteins in buffalo bull SP.

## 1. Introduction

Seminal plasma (SP) is an extremely complicated biological fluid containing electrolytes, hormones, proteins, enzymes, carbohydrates, and lipids that have an important impact on sperm cell function and its cryosurvival (1). Thus, SP is a reliable indicator of male fertility. Increased reactive oxygen species (ROS) in semen samples adversely affect sperm quality and fertility. Moreover, the high content of polyunsaturated fatty acids in buffalo bull sperm as compared to bull sperm renders them more vulnerable to oxidative stress (2, 3).

In humans, seminal fructose (4) is the main source of energy production alongside glucose in the majority of mammalian species. Glucose and fructose were found to have beneficial effects on mammalian gametes in terms of metabolizable energy and fertility potential (5). Additionally, fructose was higher in highly fertile bulls, suggesting that it could be used as a seminal biomarker for bull fertility (6). Both serum and seminal testosterone levels were positively related to sperm quality and fertility in rams (7, 8), and it promotes sperm production in bulls (9). Both LH and FSH regulate testosterone synthesis in Leydig cells and are also responsible for male fertility (9). Reproductive biologists believe that metabolic hormones play a role in spermatogenesis/steroidogenesis, and seminal concentrations of IGF-1 are positively related to stallion fertility (10). Also, the post-thaw sperm motility and viability were greatly improved by adding IGF-1 (11) to the freezing extender in buffalo bulls.

Both albumin and globulin constitute the major fraction of SP proteins, whereas non-protein nitrogen, amino acids, and peptides represent limited quantities. Proteins of SP have amphoteric properties and thus, low protein content in SP reduced its buffering capacity, and ultimately decreased sperm quality and fertility (12). Seminal plasma lipids play an important role in sperm membrane structure and function. Furthermore, there is evidence that semen volume, sperm motility, and concentration are influenced by lipid species in SP (13). SP proteins are species-specific and some of them are closely related to male fertility (14, 15). Previous research has focused on identifying and isolating specific seminal proteins that may affect buffalo sperm capacitation and/or fertilization (14, 16, 17). Previous proteomic studies on SP proteins, to the best of our knowledge, did not combine hormonal and biochemical components with proteomic analyses. As a result, the present study looks for hormonal, biochemical, and proteomic components in SP and/or serum collected from buffalo bulls of different *in vivo* fertility which could be used as markers of fertility.

## 2. Materials and methods

Except otherwise specified, the chemicals used were of high purity and procured from Merck KGaA (Darmstadt, Germany).

### 2.1. Experimental animals

A total of 14 healthy and mature Egyptian buffalo bulls (*Bubalus bubalis*, 4–5 years old) with good body condition scores were classified depending upon their conception rate (CR) obtained following insemination of 330 estrus pluriparous buffalo cows (4–8 years old) during the last two previous breeding seasons using their frozen-thawed straws into fertile (*n* = 10, CR ≥ 55%) and subfertile (*n* = 4, CR ≤ 35%) groups in line with Kumar et al. (18). All buffalo bulls were kept at Mahallet-Mousa Research Farm located in Mahallet-Mousa, Kafrelsheikh (latitude 31° 06′ N and longitude 30° 56′ E), Egypt. Animals were maintained in open yards, fed a concentrated diet combination and roughages according to the National Research Council (19) standards, with free access to clean water and mineral blocks. All animal experiments were conducted according to the ARRIVE guidelines (https://arriveguidelines.org) and approved by the Committee for Ethics in Research, Faculty of Veterinary Medicine, Kafrelsheikh University, Egypt.

### 2.2. Semen collection and evaluation

From September to November, semen samples were collected twice a week at 07:00–08:00 a.m. using an artificial vagina (with an inner sleeve temperature of 40°C) for 6 weeks (12 ejaculates/animal). Immediately after collection, the collected ejaculates were visually examined for color, consistency, and hygienic quality, and also ejaculate volume was noticed. From each ejaculate, an aliquot (200 μL) was used to determine the following sperm characteristics and kinematics:

#### 2.2.1. Sperm kinematics and viability

Sperm kinematics were determined using a computer-aided sperm motion analyzer (CASA; Hamilton Thorne, Inc., Beverly, MA, USA) system. The sperm motility was calculated with speed standards set as fast; >80 μm/s, medium; >60 μm/s, slow; >20 μm/s, and static. From each ejaculate, an aliquot was diluted (1:10) with prewarmed Tris buffer before being (5 μL) loaded into a prewarmed (37°C) Makler counting chamber to determine total motility (%), progressive motility (%), average path velocity (VAP, μm/s), straight linear velocity (VSL, μm/s), curvilinear velocity (VCL, μm/s), straightness (STR, %), linearity (LIN, %), and wobble coefficient (WOB, %) according to Kumar et al. (3). For each evaluation, eight microscopic fields were randomly selected and analyzed by the CASA system. An eosin-nigrosin-stained semen smear was examined to estimate the proportion of viable spermatozoa (20). At least 200 spermatozoa were examined under an oil immersion lens (1,000×) where sperm cells with unstained heads expressed percent sperm viability.

#### 2.2.2. Functional plasma membrane integrity

To assess the functional integrity of the sperm plasma membrane, sperm cells were subjected to a hypo-osmotic swelling test [HOST; (21)]. In brief, a prewarmed hypo-osmotic solution (1,000 μL) of 150 mOsm/kg osmolarity (fructose = 1.351 g, sodium citrate = 0.735 g dissolved in 100 mL Milli-Q water) was mixed with 100 μL semen. This sperm suspension was incubated at 37°C for at least 30 min. After incubation, an aliquot (2 μL) of this suspension was spotted onto a prewarmed clean glass slide, covered by a prewarmed coverslip (18 × 18 mm), and visualized under 400× magnification. At least 200 spermatozoa were carefully examined for the proportion of spermatozoa showing curling or swelling of their tails (% HOST-positive). The proportion of spermatozoa with abnormal tail morphology was already determined before HOST and was subtracted from the proportion of HOST-positive spermatozoa to obtain the true percentage of HOST-positive spermatozoa.

#### 2.2.3. Acrosomal membrane integrity

Fluorescein isothiocyanate-conjugated peanut agglutinin (FITC-PNA) staining technique was used to determine acrosomal membrane integrity as described in our previous research (22). Briefly, an aliquot (10 μL) of semen was fixed with 4% paraformaldehyde (Sigma Chemical Company, USA) at room temperature for 30 min and then diluted (1:10) with phosphate-buffered saline (PBS) containing 0.1% polyvinyl alcohol (Sigma Chemical Company, USA) and 0.1% polyethylene glycol (Sigma Chemical Company, USA) before being (5 μL) smeared onto a glass slide and dried on a warmed plate at 38.5°C. Spermatozoa were permeabilized by using 200 μL of 1% (v/v) Triton X-100 for 5 min at room temperature and then allowed to dry and stained with FITC-PNA (20 μg/mL; Sigma Chemical Company, USA) for 30 min in a humidified chamber in a dark place.

After incubation, stained smears were rinsed with PBS to remove unbound probes, allowed to dry, and covered with 0.22 M 1,4-diazabicyclo [2,2,2] octane (Sigma–Aldrich, Germany) dissolved in glycerol-PBS mixture (9:1) (DABCO). Stained smears were covered with a coverslip (24 mm × 50 mm) before examination with a phase-contrast microscope with fluorescence illumination (mirror unit U-MWB2: excitation filter, BP460–490, dichroic mirror DM500, and emission filter BA520IF; Olympus, Tokyo, Japan). Sperm cells with uniform, intense, and well-demarcated green fluorescent acrosomes were graded as intact-acrosomes while those exhibiting fluorescence in the anterior region only, no fluorescence in the head, or fluorescence only along the outline or acrosomal fringe were graded as damaged-acrosomes. At least 200 spermatozoa were examined in each smear to calculate the intact-acrosomes percentage.

### 2.3. Recovery of seminal plasma and blood collection

The remaining volume of ejaculate was centrifuged at 12,000 × *g* in a cooling centrifuge at 4°C for 30 min to collect SP. The supernatant (SP) was re-centrifuged at 12,000 × *g* for 10 min in a microfuge (Mittelsachsen, Saxony, Germany) to obtain clear SP. Total protein concentration (g/dL) in SP was determined with a hand-held refractometer [ATAGO, Brix 0–32%, Japan, (23)], and then SP was frozen stored at −80°C until hormonal, biochemical, and proteomic analyses. In parallel, throughout the experiment, blood samples have been drawn from the jugular vein of all buffalo bulls before ejaculation. The collected blood samples were centrifuged at 2,500 × *g* for 20 min at 4°C to separate serum, and the collected serum was also frozen stored at −80°C until hormonal and biochemical analyses.

### 2.4. Hormonal analyses

Serum and seminal assays of LH and FSH were performed by chemiluminescence through the immune-enzymatic technique using the commercial kits Beckman Coulter^®^ (Beckman Coulter, USA) and the Access^®^ equipment (Beckman Coulter, USA) according to the manufacturer's instructions. Serum and seminal total testosterone levels were measured using commercial ELISA kits (Bio Check, Foster City, CA, USA) in duplicate with a sensitivity of 0.5 ng/mL, while the intra- and inter-assay coefficients of variation (CV) were 7.7 and 9.0%, respectively. The immuno-radiometric assay kit (Immunotech SAS, Marseille Cedex, France) was used to measure serum and seminal IGF-1 levels (24), with a 2 ng/mL sensitivity and an intra-assay CV of 3.26%.

### 2.5. Biochemical analyses

Total antioxidant capacity (TAC) was estimated using an antioxidant assay kit provided by Cayman chemical company (Michigan, USA) as designated by Lone et al. (25). Seminal levels of Catalase [CAT; (26)], Glutathione peroxidase [GPx; (3)], Nitric oxide [NO; (27)], and Malondialdehyde [MDA; (3)] were calculated. Seminal fructose was measured with the colorimetric method (28), whereas the Cobas c 311/501 Analyzer (Roche Diagnostics GmbH, Mannheim, Germany) was utilized to determine the serum glucose level. Colorimetric methods were used to assess the serum and seminal amounts of total protein (29) and albumin (30). Also, serum and seminal contents of triglycerides, cholesterol, and high-density lipoproteins (HDL) were calorimetrically determined using commercial kits (Bio-Diagnosis Co., Cairo, Egypt).

### 2.6. SDS-PAGE of SP proteins

This experiment was run in four repetitions, utilizing the SP of both fertile and subfertile buffalo bulls in the same separating gel in the presence of a known standard to evaluate the relative content of different SP proteins and their molecular weight. Frozen SP was thawed and adjusted to a total protein concentration of 500 μg/25 μL before being subjected to SDS-PAGE (31). Briefly, electrophoresis sample buffer [4% (w/v) SDS, 20% (v/v) glycerol, 10% (v/v) β-mercaptoethanol, and pH 6.8] was used to dilute (1:1) SP samples then boiled for exactly 5 min, followed by centrifugation at 10,000 × *g* at 4°C for 5 min to collect the supernatants containing SP proteins.

Extracted SP proteins were separated on a 15% (w/v) polyacrylamide gel containing 0.1% (w/v) SDS at room temperature and 20 mA/gel using a mini protean III vertical slab gel electrophoresis apparatus (Bio-Rad Laboratories, Hercules, CA, USA). The separating gels were immersed for 5 min in a freshly prepared pre-fixative solution containing 20% (v/v) methanol and 7.5% (v/v) acetic acid in H_2_O before being stained with 0.1% (w/v) Coomassie brilliant blue R-250 (Oxford, Mumbai, India) at room temperature with gentle shaking for 1 h (32). Stained gels were destained in a solution of 30% (v/v) methanol and 10% (v/v) acetic acid in H_2_O overnight with gentle shaking. Finally, the gel was scanned with a scanner (HP Scanjet G3110, Hong Kong, China) and the apparent molecular weight was calculated with a broad-way dual prestained protein marker (6.5–212 kDa, New England, BioLabs, UK). The proportions of different protein fractions were also determined using an analytical system (Gel-Doc. Model-Alpha Imager TM1220; Alpha Innotech Corporation, Santa Clara, CA, USA).

### 2.7. Statistical and image analyses

The results are tabulated as mean ± SEM. A GraphPad Prism computer program version 7.0 (GraphPad Software, San Diego, CA, USA) was used to perform all statistical analyses, and a Student's *t*-test was employed to compare fertile and subfertile groups. Differences with *P* < 0.05 were considered statistically significant. Using the gel-doc system, gel images were processed to quantify the molecular weights of protein bands as well as the relative protein fractions (protein %).

## 3. Results

### 3.1. Sperm characteristics and kinematics of fertile and subfertile buffalo bulls

As shown in Table 1, the values of sperm characteristics (total motility, progressive motility, viability, intact-plasma membrane, and intact-acrosome) and sperm kinematics variables except LIN and WOB were higher (*P* < 0.01) in the fertile group than in the subfertile group.

**Table 1:** Sperm characteristics and kinematics (mean ± SEM) of fertile and subfertile buffalo bulls.

**Parameter**	**Fertile (*n* = 10)**	**Subfertile (*n* = 4)**	***P*-values**
Total motility (%)	77.60 ± 1.11^a^	68.64 ± 0.79^b^	<0.001
Progressive motility (%)	73.73 ± 0.49^a^	63.87 ± 0.54^b^	<0.001
Viability (%)	91.41 ± 1.25^a^	79.37 ± 1.19^b^	<0.001
Intact-plasma membrane (%)	74.17 ± 1.11^a^	59.67 ± 1.73^b^	<0.001
Intact-acrosome (%)	92.83 ± 0.79^a^	84.83 ± 0.79^b^	<0.001
VAP (μm/s)	75.60 ± 0.85^a^	68.31 ± 0.95^b^	<0.001
VSL (μm/s)	27.72 ± 0.62^a^	22.02 ± 0.84^b^	<0.001
VCL (μm/s)	94.57 ± 0.93^a^	79.21 ± 1.24^b^	<0.001
STR (%)	36.65 ± 0.47^a^	32.21 ± 1.06^b^	<0.01
LIN (%)	29.35 ± 0.92^a^	27.88 ± 1.37^a^	0.3921
WOB (%)	80.03 ± 1.70^a^	86.38 ± 2.55^a^	0.0651

Means bearing different superscripts within the same row were different at P < 0.05.

VAP, Average path velocity; VSL, Straight linear velocity; VCL, Curvilinear velocity; STR, Straightness; LIN, Linearity; WOB, Wobble.

### 3.2. Hormonal and biochemical analyses

Both seminal and serum levels of FSH, LH, testosterone, and IGF-1 were greater (*P* < 0.01) in the fertile group than in the subfertile group, as shown in Tables 2, 3. Also, seminal TAC, CAT, GPx, and NO were higher (*P* < 0.05) in the fertile group. Likewise, seminal fructose, total protein, albumin, triglycerides, cholesterol, and HDL were greater (*P* < 0.01) in the fertile group compared with the subfertile group (Table 2). On the contrary, seminal MDA was greater (*P* < 0.001) in the subfertile (1.56 ± 0.08 μM/mL) group than in the fertile (0.96 ± 0.05 μM/mL) group as presented in Table 2. Serum levels of glucose, total protein, albumin, triglycerides, cholesterol, and HDL were comparable (*P* ≥ 0.05) between the two fertility groups as given in Table 3.

**Table 2:** Seminal fertility biomarkers (mean ± SEM) of fertile and subfertile buffalo bulls.

**Assay**	**Parameter**	**Fertile (*n* = 10)**	**Subfertile (*n* = 4)**	***P*-values**
Hormonal	FSH (mUI/mL)	0.37 ± 0.03^a^	0.19 ± 0.01^b^	<0.001
	LH (mUI/mL)	0.28 ± 0.02^a^	0.11 ± 0.01^b^	<0.001
	T (ng/mL)	0.53 ± 0.02^a^	0.38 ± 0.02^b^	<0.001
	IGF-1 (ng/mL)	57.67 ± 1.71^a^	50.33 ± 1.48^b^	<0.01
Biochemical	TAC (Mm)	2.28 ± 0.07^a^	1.81 ± 0.05^b^	<0.001
	CAT (u/mL)	38.17 ± 1.04^a^	30.33 ± 1.19^b^	<0.001
	GPX (nmol/min/mL)	23.00 ± 0.89^a^	19.50 ± 0.76^b^	<0.05
	NO (mmol/L)	35.50 ± 1.23^a^	25.50 ± 0.85^b^	<0.001
	MDA (μM/mL)	0.96 ± 0.05^b^	1.56 ± 0.08^a^	<0.001
	Fructose (mg/dL)	322.00 ± 3.92^a^	277.30 ± 2.30^b^	<0.001
	Total protein (g/dL)	6.33 ± 0.09^a^	4.00 ± 0.37^b^	<0.001
	Albumin (g/dL)	2.36 ± 0.07^a^	1.36 ± 0.06^b^	<0.001
	Triglycerides (mg/ dL)	58.83 ± 1.47^a^	50.50 ± 1.29^b^	<0.01
	Cholesterol (mg/dL)	117.70 ± 2.38^a^	78.17 ± 1.89^b^	<0.001
	HDL (mg/dL)	30.83 ± 0.95^a^	22.67 ± 1.09^b^	<0.001

Means bearing different superscripts within the same row were different at P < 0.05.

T, Testosterone; TAC, Total antioxidant capacity; CAT, Catalase enzyme; GPx, Glutathione peroxidase, NO, Nitric oxide; MDA, Malondialdehyde; HDL, High-density lipoproteins.

**Table 3:** Serum fertility biomarkers (mean ± SEM) of fertile and subfertile buffalo bulls.

**Assay**	**Parameter**	**Fertile (*n* = 10)**	**Subfertile (*n* = 4)**	***P*-values**
Hormonal	FSH (mUI/mL)	0.70 ± 0.02^a^	0.32 ± 0.02^b^	<0.001
	LH (mUI/mL)	0.52 ± 0.01^a^	0.22 ± 0.01^b^	<0.001
	T (ng/mL)	1.39 ± 0.04^a^	0.85 ± 0.01^b^	<0.001
	IGF-1 (ng/mL)	1678 ± 28.85^a^	1356 ± 14.19^b^	<0.001
Biochemical	Glucose (mg/dL)	69.83 ± 1.79^a^	67.67 ± 1.02^a^	0.319
	Total protein (g/dL)	9.56 ± 0.09^a^	9.48 ± 0.09^a^	0.542
	Albumin (g/dL)	3.70 ± 0.07^a^	3.59 ± 0.02^a^	0.194
	Triglycerides (mg/dL)	62.33 ± 1.71^a^	61.33 ± 1.09^a^	0.631
	Cholesterol (mg/dL)	176.80 ± 2.26^a^	177.70 ± 1.63^a^	0.770
	HDL (mg/dL)	56.50 ± 0.76^a^	56.50 ± 0.96^a^	0.999

Means bearing different superscripts within the same row were different at P < 0.05.

T, Testosterone; HDL, High-density lipoproteins.

### 3.3. Protein profiles and protein fractions of SP collected from fertile and subfertile buffalo bulls

**Figure 1:**
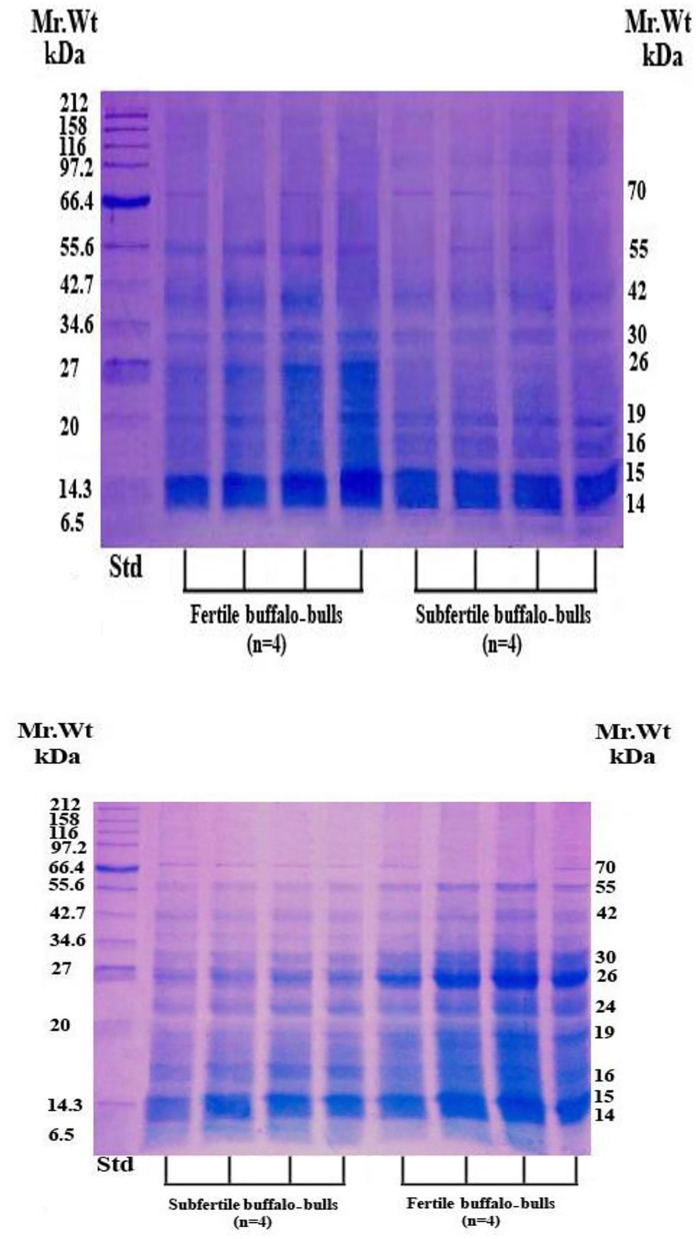
SDS-PAGE of SP proteins in fertile and subfertile buffalo bulls. Bulls were classified into fertile and subfertile groups based on their conception rates. SP was collected by centrifugation (12,000 × *g* for 30 min) of the collected semen samples. Reduction, denaturation, and separation of SP proteins on 15% polyacrylamide gel were performed in the presence of a broad-way (6.5–212 kDa) prestained protein marker. Separated protein bands were detected by Coomassie brilliant blue staining. Std, Standards; kDa, Kilo Dalton; Mr. Wt, Molecular Weight.

The SP protein profile (kDa) and their protein fractions (%) of fertile and subfertile buffalo bulls are represented in Table 4 and Figure 1. Gel analysis revealed 10 (70, 55, 42, 30, 26, 24, 19, 16, 15, and 14 kDa) protein bands with a molecular weight ranging from 14 to 70 kDa were identified in both fertile and subfertile buffalo bulls. Almost all the detected bands were larger and denser in the fertile group compared with the subfertile group as illustrated in Figure 1. It is worth mentioning that protein bands of 14, 15, 26, 30, and 55 kDa were more predominant in the fertile group compared to the subfertile one. Additionally, the protein % of 55, 30, and 26 kDa protein bands was considerably greater (*P* < 0.001) in the fertile group, whereas, the protein % of the 16 kDa protein band was considerably greater (*P* < 0.001) in the subfertile group as presented in Table 4.

**Table 4:** Seminal plasma proteins profiles (kDa) and protein fractions (%) of fertile and subfertile buffalo bulls.

**Estimated molecular** **weight (kDa)**	**Candidate protein** **(molecular weight, kDa)**	**% Fraction**		***P*-values**
		**Fertile**	**Subfertile**	
70	Albumin (71), Clusterin precursor (70)	1.74 ± 0.14^a^	1.75 ± 0.05^a^	0.9960
55	Osteopontin (55)	5.00 ± 0.13^a^	1.93 ± 0.16^b^	<0.001
42	Clusterin (40) or Ecto-ADP-ribosyltransferase 5 (43)	3.67 ± 0.09^a^	3.44 ± 0.25^a^	0.4059
30	BSP-30 kDa (28–30)	5.13 ± 0.23^a^	2.84 ± 0.10^b^	<0.001
26	Prostaglandin D synthase	5.30 ± 0.19^a^	1.87 ± 0.25^b^	<0.001
24	TIMP-2 (25,26)	4.15 ± 0.09^a^	3.83 ± 0.18^a^	0.1573
19	Novel protein	2.72 ± 0.09^a^	2.78 ± 0.12^a^	0.7194
16	BSP-A3 (16.5)	1.61 ± 0.05^b^	3.96 ± 0.18^a^	<0.001
15	BSP-A2 (15,16)	13.00 ± 0.26^a^	12.89 ± 0.14^a^	0.7200
14	BSP-A1 (15) or Spermadhesin (14)	11.38 ± 0.26^a^	10.97 ± 0.22^a^	0.2758

Means bearing different superscripts within the same row were different at P < 0.05.

kDa, Kilo Dalton.

## 4. Discussion

Male fertility is a multifactorial trait depending upon several factors, including animal behavior and physical fitness, as well as semen quality and biochemical and/or hormonal components of SP (33). As we expected, in the present study, the proportion of sperm motility and viability, and almost all sperm kinematics were significantly higher in fertile buffalo bulls compared with subfertile animals. This is likely attributed to normal testicular anatomy, histology, and function in fertile animals, which backed the findings of Singh et al. (34), who found that the proportion of motile spermatozoa in highly fertile bulls was higher (*P* < 0.001) than that of low fertile bulls. Further, our findings indicated that the proportions of intact-acrosome and intact-plasma membrane were greater in the fertile animals compared to the subfertile ones, which increased oocyte penetration and enhanced the fertilization process (35).

Oxidative stress is among the most implicated causes of low semen quality and has a prolonged history of being linked to male subfertility (36). This oxidative stress increases sperm cell abnormalities, loss of membrane integrity, DNA damage, and enzyme inactivation, leading to subfertility and/or infertility (37). Antioxidants in the SP or on spermatozoa are the major defense mechanisms against oxidative stress by minimizing the liberation of free radicals. As a result, they enhance sperm motility and might be beneficial in the treatment of male subfertility and/or infertility (38). Herein, fertile buffalo bulls had greater seminal TAC, CAT, GPx, and NO and lower MDA content than subfertile buffalo bulls, emphasizing the notion that MDA is a cytotoxic aldehyde and its elevation in SP has negative impacts on the quality and fertility of sperm cells due to its action on lipid membrane structures (39). In line with our results, Barranco et al. (40) reported that antioxidants have a favorable correlation with the fertility index, which may prevent oxidative damage to the sperm plasma membrane and might be used as a strong fertility estimate in boars. This could explain the higher seminal values of TAC, CAT, GPx, and NO of fertile buffalo bulls obtained in the current trial.

IGF-1 plays an important role in energy metabolism and improves the metabolic activity of buffalo bull spermatozoa through increasing intracellular calcium ion concentration, which results in increased progressive sperm motility (41). Our results indicated that fertile buffalo bulls had higher seminal and serum IGF-1 concentrations, which is partially consistent with Kumar et al. (42), who found that serum IGF-1 concentrations in fertile buffalo bulls were higher than those of subfertile animals and were positively correlated with sperm concentration, mass motility, and fertility. Our findings, On the other hand, are inconsistent with Kumar et al. (42) who found no difference in seminal IGF-1 levels between fertile and subfertile buffalo bulls. This might be due to our study having a higher number of fertile (10 *vs*. 5) and subfertile (4 *vs*. 2) buffalo bulls than Kumar et al. (42). Thus, quantification of seminal and/or serum IGF-1 concentration is recommended to predict sperm cell functions (43, 44).

In our trial, fertile buffalo bulls had greater levels of serum and seminal testosterone compared to subfertile animals. This is likely due to testosterone regulating testicular function, particularly Sertoli cell function, and also has a crucial role in the process of spermatogenesis (45). Recently, it has been proven that seminal testosterone level is positively associated with sperm cell concentration and the proportion of motile spermatozoa in fertile bulls (46). Both FSH and LH regulate testosterone synthesis and sustain proper spermatogenesis, sperm vitality, and density (47). This might explain why the fertile group had greater seminal and serum FSH and LH levels than the subfertile group. The low LH concentration in the subfertile group may not activate the Leydig cells, resulting in low serum and seminal testosterone levels. Moreover, low FSH levels impair Sertoli cell activity, leading to subfertility or even infertility (48). Based on these findings, it is plausible to obtain greater levels of FSH and LH in either the serum or SP of fertile buffalo bulls.

Seminal fructose is produced *via* the conversion of glucose with aldose reductase into sorbitol in the vesicular glands, which is testosterone-dependent, and eventually, sorbitol dehydrogenase reduces sorbitol into fructose (49). Our results indicated that seminal fructose was greater in the fertile group than in the subfertile one; this supports the fact that seminal fructose plays an important role in the metabolic activity of sperm cells, where sperm cells consume fructose to produce ATP to maintain their motility (50).

Our findings reveal that the fertile group has higher seminal total protein and albumin content in comparison with the subfertile group. It is well-known that the seminal protein fraction constitutes the amphoteric characteristic of SP and represents the main component of its buffering activity (51). Also, seminal albumin plays a crucial role in sperm motility, capacitation, and acrosome reaction by acting as a cholesterol acceptor, leading to cholesterol efflux from the sperm plasma membrane (52), which is an initial and essential step in the fertilization cascade. These aforementioned findings might be the reason for the low semen quality and/or low fertility of subfertile buffalo bulls.

Cholesterol has a pivotal role in sperm motility, capacitation, acrosome reaction, and fertility (53). In the current study, the fertile group had a higher seminal cholesterol level (117.70 ± 2.38 mg/dL) than the subfertile (78.17 ± 1.89 mg/dL) group, which is consistent with the findings of El-Sayed et al. (54), who reported that seminal cholesterol level was positively correlated with ejaculate volume, total sperm cell count per ejaculate, sperm motility, viability, as well as the integrity of plasma and acrosomal membranes in buffalo bulls. Similarly, seminal triglyceride content was greater in the fertile (58.83 ± 1.47 mg/dL) buffalo bulls because they are one of the main energy substrates available for sperm cell metabolism; thus, low triglyceride levels might lead to insufficient energy, low sperm motility, and subfertility (55). Moreover, seminal triglycerides and HDL levels in buffalo bulls were also positively associated with sperm concentration, the total number of sperm cells per ejaculate, and acrosomal integrity (54).

In our study, 10 protein bands were detected in SP collected from either fertile or subfertile buffalo bulls but those of 14, 15, 26, 30, 42, and 55 kDa were predominant in the fertile animals. Protein bands of 70, 55, 42, 26, and 24 kDa may correspond to albumin or clusterin precursor, osteopontin, clusterin, or ecto-ADP-ribosyltransferase 5, prostaglandin D synthase, tissue inhibitor metalloproteinase-2 (TIMP-2), respectively as summarized in Table 4 (56, 57). Protein bands of 30, 16, 15, and 14 kDa may correspond to bovine seminal plasma protein-30 kDa (BSP-30 kDa), BSP-A3, BSP-A2, and BSP-A1 or spermadhesin, respectively (58).

Notably, the protein % of 55, 30, and 26 kDa protein bands was higher in the fertile group (Table 4), indicating that these protein bands are related to *in vivo* fertility at least in Egyptian buffalo bulls. In accord with Killian et al. (59), we found that the protein % of the 16 KDa protein band was higher in subfertile animals, indicating that this protein band might be used as a diagnostic for male subfertility. Based on the findings of Gwathmey et al. (60) who reported that all BSPA1/A2, BSP-A3, and BSP-30 kDa are the major proteins in bovine SP which play substantial roles in animal fertility, and also results of the current study revealed that protein bands of 14, 15, 26 and 30 kDa were denser and larger in fertile buffalo bulls, suggesting that these proteins are fertility-associated at least in buffalo bulls.

It is well-known that osteopontin is a 55 kDa acidic SP protein that has a critical role in sperm oocyte encounter and the early stages of embryo development (61) which might be one of the possible reasons for the difference in the *in vivo* fertility of the two animal groups (fertile *vs*. subfertile). Furthermore, a protein band of 26 kDa might be prostaglandin D synthase that is localized in the apical ridge of the sperm acrosome (62) and indirectly helps the male genital organs in uptaking retinoids, essential for growth, differentiation, and spermatogenesis (63).

## 5. Conclusions

Seminal TAC, CAT, GPx, NO, and fructose, as well as seminal and/or serum FSH, LH, testosterone, and IGF-1, are all fertility-associated biomarkers. Seminal levels of total protein, albumin, triglycerides, cholesterol, and HDL can also be used to distinguish fertile buffalo bulls from subfertile buffalo bulls. Furthermore, protein bands of 26, 30, and 55 kDa in buffalo bull SP may represent fertility-associated proteins that require further investigation in the future.

## Data availability statement

The datasets presented in this study can be found in online repositories. The names of the repository/repositories and accession number(s) can be found in the article/supplementary material.

## Ethics statement

The animal study was reviewed and approved by the ARRIVE guidelines (https://arriveguidelines.org) and approved by the Committee for Ethics in Research, Faculty of Veterinary Medicine, Kafrelsheikh University, Egypt.

## Author contributions

Conceptualization, methodology, investigation, data curation, writing, review, and editing of the manuscript, supervision, formal analysis, and visualization were performed by EA, AA, FS, KK, TA, and WE. AA, FS, and WE drafted the manuscript, performed the statistical analysis, and supervised by EA. All authors read and approved the final manuscript.
